# Supportive care needs and quality of life among cancer patients in China: A cross-sectional study

**DOI:** 10.1371/journal.pone.0331149

**Published:** 2025-08-28

**Authors:** Bingshuang Wang, Xinyan Hu, Wanzhen Ding, Huimin Guo, Xiaoliang Ren

**Affiliations:** 1 School of Nursing, Hebei Medical University, Shijiazhuang, Hebei, China; 2 Department of Medical Oncology, the Fourth Hospital of Hebei Medical University, Shijiazhuang, Hebei, China; 3 Department of Blood Transfusion, the Fourth Hospital of Hebei Medical University, Shijiazhuang, Hebei, China; Mae Fah Luang University School of Anti Aging and Regenerative Medicine, THAILAND

## Abstract

**Introduction:**

In this study, the factors associated with unmet supportive care needs (SCNs) were examined, and their correlation with quality of life (QoL) among cancer patients was explored.

**Methods:**

This cross-sectional study included 137 cancer patients who were recruited via convenience sampling from an oncology department at a tertiary cancer hospital in China. Three assessment instruments were employed herein: a general information questionnaire, the Chinese version of the Supportive Care Needs Survey short form (SCNS-SF34-C), and the functional subscale of the European Organization for Research and Treatment of Cancer Quality of Life Questionnaire-Core 30 (EORTC QLQ-C30).

**Results:**

Among the five domains of the SCNS-SF34-C, the health system and information domain had the highest mean score (M = 2.74; SD = 0.75), followed by the psychological domain (M = 2.36; SD = 0.85). Five factors were associated with the unmet SCNs of cancer patients: sex, educational background, disease awareness, smoking status, and drinking habits (all *p* < 0.05). Moreover, participants with unmet psychological, physical and daily living needs had significantly lower QoL scores in all functional and symptom domains (all *p* < 0.05).

**Conclusions:**

Chinese cancer patients face notable unmet needs in the health system and information domain. Future studies should focus on designing individualized interventions to improve supportive care among cancer patients and to enhance their QoL.

## Introduction

Cancer is a leading cause of death worldwide. The International Agency for Research on Cancer reported that there were 19.29 million new cancer cases and 9.96 million cancer-related deaths worldwide in 2020 [1]. In China, cancer is responsible for 30.1% of deaths (3 million); furthermore, China accounts for 23.7% of all new cancer cases globally (4.57 million). Recent global projections indicate that there will be approximately 35 million new cancer cases recorded by the year 2050.

Cancer treatment poses a range of physical, psychological, social, and spiritual challenges for patients, frequently leading to a reduced quality of life (QoL). Supportive care is a patient-centered strategy that connects patients with services to address their informational, practical, and emotional needs throughout cancer treatment. Several studies have indicated that cancer patients often face unmet supportive care needs (SCNs), which may lead to poor treatment compliance and decreased mental health [2,3]. In a position paper, the European Society for Medical Oncology highlighted the gap between the needs of cancer patients and the actual care they receive [4]. Unmet SCNs can compromise cancer patients’ ability to manage their illness effectively, thereby intensifying their emotional and psychological burdens, negatively impacting their QoL and potentially worsening their disease outcomes [5]. Numerous studies have demonstrated links among unmet SCNs, increased symptom burden, and reduced QoL [6,7]. Addressing these unmet needs significantly improves patients’ overall well-being [8].

Assessing the unmet needs of cancer patients and analyzing their influencing factors can provide such patients with more effective support and intervention. In this respect, nurses play a critical role not only in identifying changing patient needs but also in providing timely interventions during each treatment period based on patients’ unmet needs [9]. This has significant research implications for improving the quality of medical services, optimizing resource allocation, supporting policy-making, promoting the development of clinical research, and addressing caregiver needs.

To date, studies examining the unmet SCNs of cancer patients have often focused on specific cancer types, such as breast [2], colorectal [10], and other common cancers [8,11]. There is no consensus on the commonality of unmet SCNs across different cancer types and populations. Studies have shown that addressing unmet SCNs in cancer patients can effectively improve their QoL [12]. Identifying unmet SCNs can enhance cancer patients’ psychological well-being and QoL, and inform clinical practice [13]. Understanding the current unmet SCNs of patients can serve as a screening tool, enhancing medical services and advancing the development of clinical care decisions and interventions [14]. Therefore, the current study aimed to (1) assess unmet SCNs, (2) identify the factors associated with unmet SCNs, and (3) analyze the relationship between SCNs and QoL in cancer patients in China. These results will potentially provide guidance for the development of comprehensive support strategies.

## Methods

### Participants

This cross-sectional study included cancer patients recruited from an oncology department at a tertiary cancer hospital in China from July to August 2023. Before the questionnaires were distributed, informed consent was obtained from the participants after a thorough explanation of the study’s purpose and significance was provided. For participants with low literacy or those who required assistance due to age (e.g., completing the questionnaire), the investigators provided clarifications for each item and assisted in its completion, ensuring data quality and reliability. The proposed sample size ranged from five to ten times the number of items on the main scale of this study [15]. This study examined 20 key variables: 9 demographic variables (e.g., age, sex), 5 variables from the SCNS-SF34-C questionnaire, and 6 variables from the EORTC QLQ-C30 questionnaire. It was estimated that 100–200 participants were needed. We ultimately approached 150 patients. The inclusion criteria were as follows: (1) confirmed cancer diagnosis via pathology or imaging, (2) willingness to participate in the study, (3) aged 18 years or older, and (4) ability to communicate and complete the research questionnaire with investigator assistance. The exclusion criteria were as follows: (1) recent exposure to other major stressful events and (2) the presence of mental disorders.

### Ethical approval

The study was approved by the Ethics Committee of Hebei Medical University (protocol no. 2020206). All participants provided written informed consent. For participants with limited literacy and those who required assistance due to age-related factors, the investigators read each questionnaire item and facilitated its completion to ensure data quality and reliability.

### Measures

#### Sociodemographic and clinical characteristics.

The following sociodemographic data were collected from patients using general information questionnaires: age, sex, occupation, marital status, educational background, disease awareness, monthly income, drinking habits, and smoking habits. Clinical data, including primary cancer type and treatment, were obtained from electronic medical records. The data were subsequently reviewed by the clinical staff (research team member) to ensure completeness and consistency. Completeness checks involved line-by-line comparison of questionnaires with medical records to identify missing fields; gaps were filled via telephone or supplementary files. Consistency checks involved cross-validating discrepancies between sources (e.g., questionnaire “no alcohol use” vs. record “alcoholic liver disease”) and correcting implausible values (e.g., verifying an age of 150 years via chart review or patient contact).

#### Supportive care needs.

The Chinese version of the Supportive Care Needs Survey short form (SCNS-SF34-C) is a self-report questionnaire that assesses the perceived needs of cancer patients across five domains: psychological (10 items), health system and information (11 items), physical and daily living (5 items), patient care and support (5 items), and sexuality (3 items) [16,17]. The respondents indicate their level of need for help over the past month with respect to their cancer diagnosis via a five-point response scale: 1 (no need, not applicable), 2 (no need, satisfied), 3 (low need), 4 (moderate need), and 5 (high need). Higher scores represent greater need. A rating of 3 or higher was regarded as an unmet need [11,18]. The SCNS-SF34-C was translated by AU et al. [19] and has shown good internal consistency, with Cronbach’s alpha coefficients exceeding 0.7 for all the domains. The SCNS-SF exhibits acceptable reliability and validity, and the SCNS-SF34 has been validated in Chinese cancer patients [17,19,20].

#### Quality of life.

The European Organization for Research and Treatment of Cancer Quality of Life Questionnaire-Core 30 (EORTC QLQ-C30) is a commonly used tool for measuring QoL in cancer patients [21]. This questionnaire consists of 30 questions, including ﬁve functioning scales (physical, role, cognitive, emotional, and social), three symptom scales (fatigue, pain, and nausea/vomiting), a global QoL scale, and six single items (dyspnea, insomnia, appetite loss, constipation, diarrhea, and financial difficulties). All the items are scored on a four-point scale (1 = not at all, 2 = a little, 3 = quite a bit, and 4 = very much), except for two items on the global QoL scale (Q29 and Q30), which use a seven-point scale (1 = very poor to 7 = excellent) [22]. A higher score on the functioning scale represents a higher level of functioning, and a higher score on the global health status scale represents a greater QoL. Conversely, higher scores on the symptom scale indicate more severe symptoms. Its reliability and concurrent and criterion validity have been repeatedly demonstrated [23,24].

### Statistical analysis

Statistical analyses were conducted via the Statistical Package for Social Sciences (SPSS), version 23.0 (IBM Corp., Armonk, NY, USA). Descriptive statistics were used to summarize the participants’ demographic characteristics. Categorical data were expressed as counts (n) or percentages (%), and quantitative data as mean with standard deviation (SD). *T* tests (two groups) and one-way analysis of variance (ANOVA) (above two groups) were used to assess differences in demographic and clinical characteristics among SCNs. Spearman’s correlation analysis was performed to examine the relationships between SCNs and various domains of QoL. Unmet needs were defined as item mean scores of 3 or higher [11,18], and this study compared QoL between groups with met and unmet needs. Statistical significance was set at **p* *< 0.05.

## Results

### Participant characteristics

Table 1 shows the sociodemographic and medical characteristics of the participants. Among the 150 participants, 137 completed the questionnaires (response rate: 91.3%). The average age of the participants was 57.48 years (SD = 10.80). In total, 98.54% of the participants were married, and 57.96% had a middle school education. The majority of the participants were farmers (n = 68, 49.64%) and earned an income of less than 1000 Chinese yuan (CNY) per month (n = 58, 42.32%). The most common primary cancer types were digestive system cancer (n = 50, 36.50%) and lung cancer (n = 44, 32.12%).

**Table 1:** Participant sociodemographic and clinical characteristics (n = 137).

Variables	n (*%*)
Age (years), mean (SD)	57.48 (10.80)
< 60	75 (54.74)
≥ 60	62 (45.25)
Sex	
Male	70 (51.09)
Female	67 (48.91)
Occupation	
Farmer/Peasant	68 (49.64)
Having a formal job	17 (12.41)
Freelancer	22 (16.06)
Retired	30 (21.89)
Education	
≤ Primary school	36 (26.28)
Middle school	52 (37.96)
High school	28 (20.44)
≥ University	21 (15.32)
Marital status	
Married	135 (98.54)
Unmarried	2 (1.46)
Disease awareness	
Lack of understanding	4 (2.92)
Partial understanding	95 (69.34)
Complete understanding	38 (27.74)
Monthly income, ¥	
< 1000	58 (42.34)
1000–2000	32 (23.36)
2000–3000	35 (25.55)
> 3000	12 (8.75)
Primary cancer type	
Digestive system cancer	50 (36.50)
Lung cancer	44 (32.12)
Breast cancer	20 (14.60)
Other^*^	23 (16.78)

n = number of included patients. SD = standard deviation.

* Urologic cancer, gynecological cancer, head and neck cancer.

### Supportive care needs

Table 2 shows the SCNs of the participants. The average score for the items on the SCNS-SF34-C was 2.34 (SD = 0.55). Among the SCNS-SF34-C domains, the health system and information domain had the highest mean score (M = 2.74; SD = 0.75), followed by the psychological domain (M = 2.36; SD = 0.85). The sexuality domain had the lowest mean score (M = 1.10; SD = 0.33).

**Table 2:** Supportive care needs of the participants (n = 137).

Domain	Number of items	Domain total score	Item average score
		M (SD)	M (SD)
Health systems and information	11	30.18 (8.29)	2.74 (0.75)
Psychological	10	23.62 (8.53)	2.36 (0.85)
Patient care and support	5	11.63 (2.83)	2.33 (0.57)
Physical and daily living	5	10.73 (3.94)	2.15 (0.79)
Sexuality	3	3.30 (0.98)	1.10 (0.33)

n = number of included patients, M = Mean, SD = standard deviation.

Table 3 presents the top 10 items from the SCNS-SF34-C with the highest scores. Among these top-ranking items, six were from the health system and information domain, three were from the psychology domain, and one was from the patient care and support domain.

**Table 3:** Top 10 items with the highest scores on the SCNS-SF34-C (n = 137).

Rank	Items	Score	Domain
		M (SD)	
1	Having one member of hospital staff with whom you can talk to about all aspects of your condition, treatment, and follow-up	3.43 (1.24)	HSI
2	Being treated like a person not just another case	3.28 (1.31)	HSI
3	Having access to professional counseling (e.g., psychologist, social worker, counselor, nurse specialist) if you, family, or friends need it	3.26 (1.42)	HSI
4	Being treated in a hospital or clinic that is as physically pleasant as possible	3.19 (1.11)	HSI
5	Hospital staff acknowledging, and showing sensitivity to, your feelings and emotional needs	3.03 (1.30)	PCS
6	Being informed about cancer which is under control or diminishing (that is, remission)	2.77 (1.23)	HSI
7	Being informed about things you can do to help yourself to get well	2.73 (1.08)	HSI
8	Feelings about death and dying	2.50 (1.18)	PSY
9	Uncertainty about the future	2.47 (1.11)	PSY
10	Keeping a positive outlook	2.45 (1.01)	PSY

n = number of included patients. M = Mean. SD = standard deviation.

Abbreviations: HIS, Health systems and information; PCS, Patient care and support; PSY, Psychological.

Table 4 presents the factors associated with SCNs among cancer patients. Factors such as sex, educational background, disease awareness, smoking status, and alcohol consumption were associated with unmet SCNs across various domains, including physical and daily living, health system and information, psychological, and sexuality. Notably, compared with male patients, female patients needed more supportive care for physical and daily living needs (**p* *= 0.047). Significant disparities in health system and information needs were observed among patients with different educational backgrounds (**p* *= 0.042), with those with a middle school education reporting the highest needs. Moreover, the study revealed that patients’ physical and daily living needs significantly differed based on their level of disease awareness (*p = *0.016), with patients lacking awareness showing the highest needs. Furthermore, smokers had lower physical and daily living needs (**p* *= 0.027), whereas patients who stopped drinking had greater sexual needs (**p* *= 0.033).

**Table 4:** Associations between general characteristics and supportive care needs among participants.

	PDL	PSY	SEX	PCS	HSI	Total
	M (SD)	M (SD)	M (SD)	M (SD)	M (SD)	M (SD)
Sex						
Male	10.09 (3.74)	22.23 (7.73)	3.37 (1.18)	11.60 (2.91)	29.80 (8.05)	77.09 (17.43)
Female	11.42 (4.05)	25.07 (9.12)	3.22 (0.71)	11.66 (2.77)	30.57 (8.57)	81.94 (19.69)
*t*	−2.000	−1.974	0.880	−0.117	−0.540	−1.529
*p*	0.047*	0.050	0.381	0.907	0.590	0.128
Education						
Primary school and below	11.28 (4.34)	24.53 (9.30)	3.25 (0.81)	11.00 (3.04)	28.28 (8.15)	78.33 (19.83)
Middle school	10.62 (3.27)	24.23 (7.59)	3.37 (1.10)	12.10 (2.07)	32.60 (7.83)	82.90 (15.77)
High school	9.36 (3.83)	20.25 (8.32)	3.43 (1.26)	11.39 (2.90)	28.07 (7.91)	72.50 (18.10)
Undergraduate and above	11.95 (4.55)	25.05 (9.07)	3.05 (0.22)	11.86 (3.84)	30.24 (9.07)	82.14 (22.25)
*F*	2.104	1.917	0.728	1.177	2.818	2.129
*p*	0.103	0.130	0.537	0.321	0.042*	0.100
Disease awareness						
Lack of understanding	16.25 (5.25)	33.75 (10.05)	3.50 (0.58)	12.00 (1.83)	31.25 (6.80)	96.75 (23.17)
Partial understanding	10.49 (3.78)	23.18 (7.65)	3.22 (0.85)	11.76 (2.99)	30.61 (8.82)	79.26 (18.52)
Complete understanding	10.76 (3.88)	23.66 (9.93)	3.47 (1.27)	11.26 (2.51)	28.97 (7.00)	78.13 (18.20)
*F*	4.296	3.039	0.987	0.446	0.560	1.841
*p*	0.016*	0.051	0.375	0.641	0.572	0.163
Smoking status						
Smoking	6.60 (1.82)	21.40 (8.85)	3.00 (0.00)	12.40 (2.51)	25.20 (4.32)	68.60 (15.31)
Non-smoking	11.13 (3.87)	24.13 (8.75)	3.24 (0.85)	11.69 (2.59)	30.99 (8.61)	81.19 (19.08)
Quitting smoking	10.21 (4.01)	22.47 (7.89)	3.50 (1.33)	11.32 (3.52)	28.56 (7.37)	76.06 (17.27)
*F*	3.702	0.652	1.098	0.405	2.052	1.857
*p*	0.027*	0.523	0.337	0.668	0.132	0.160
Alcohol consumption						
Drinking	5.50 (0.71)	29.50 (6.36)	3.00 (0.00)	14.50 (2.12)	28.00 (2.83)	80.50 (12.02)
Non-drinking	10.92 (3.89)	23.82 (8.70)	3.18 (0.61)	11.60 (2.53)	30.50 (8.51)	80.02 (18.88)
Quitting drinking	10.5 (4.05)	22.68 (8.12)	3.68 (1.63)	11.53 (3.62)	29.35 (7.87)	77.74 (18.61)
*F*	1.966	0.709	3.504	1.052	0.308	0.191
*p*	0.144	0.494	0.033*	0.352	0.735	0.826

M = Mean. SD = standard deviation. t = t-value. F = F-value. p = p-value; a value below 0.05 is considered to be statistically significant.

*p < 0.05.

Abbreviations: PDL: Physical and daily living; PSY: Psychological; SEX: Sexuality; PCS: Patient care and support; HIS: Health systems and information.

### Associations between SCNs and QoL

S1 Table shows the differences in QoL, including global QoL, functional, and symptom domains, among groups with met and unmet needs. Individuals with unmet psychological, physical and daily living needs had lower scores for all functional domains (*p* < 0.01) and global QoL (*p* < 0.01) and higher scores for nearly all symptom domains (all *p* < 0.05), except for constipation and nausea/vomiting. Specific symptoms such as pain (*p* = 0.011), insomnia (*p* = 0.016), and financial difficulties (*p* = 0.046) were observed in individuals with unmet needs in the health system and information domain, who also reported lower levels of emotional (*p* = 0.008) and social (*p* = 0.005) functioning. Unmet patient care and support needs were associated with impaired emotional function (*p* = 0.001), whereas unmet sexual needs were associated with increased dyspnea (*p* = 0.008), pain (*p* < 0.001), fatigue (*p* = 0.014), insomnia (*p* = 0.039), appetite loss (*p* = 0.043), and nausea/vomiting (*p* = 0.025).

## Discussion

### SCNs of cancer patients

This study revealed that the most prevalent unmet needs were in the health system and information domain, which is consistent with previous studies conducted in China [25–27]. This trend was consistent with studies performed in other Asian countries/regions [28–30]. However, Western countries often report that patient care and support, as well as psychological needs, are the most frequently unmet supportive care needs [31]. This divergence may be influenced by Eastern cultural norms, where individuals may hesitate to seek health information from healthcare providers because of concerns about work-related responsibilities. In several East-Asian societies, hierarchical respect toward authority remains a deeply ingrained norm. This norm suppresses patient-initiated questions during clinical encounters because patients perceive direct inquiry as confrontational or disrespectful to physicians [32]. A second, complementary mechanism is time sensitivity: patients who are conscious of clinicians’ heavy caseloads voluntarily withhold questions to conserve scarce consultation time [33]. A qualitative study revealed that many patients lack experience in selecting treatments and may struggle to understand their doctor’s recommendations [34]. Consequently, they require comprehensive information about treatment options to make informed decisions. Previous research by Cuthbert et al. [35] emphasized the importance of cancer patients engaging in communication with medical teams and being informed about available resources and the unique challenges associated with different tumor types. Therefore, tailored health education programs should be developed to address the specific needs of individual patients.

In this study, the psychological domain of the SCNs ranked second. The item with the highest mean score was “feelings about death and dying”, followed by “uncertainty about the future” and “keeping a positive outlook” in the psychological domain. Various studies focusing on specific diseases, such as nervous system neoplasms [36], breast cancer [37], and young cancer patients [5,38], have consistently highlighted the importance of psychological support in patient care. This underscores the necessity for healthcare providers to prioritize the mental well-being of cancer patients by providing suitable support, educating them on therapies, and utilizing positive communication strategies [39]. These interventions have the potential to improve patients’ mental health, reduce psychological distress, and cultivate a positive mindset throughout their treatment process.

The lowest scores were recorded in the domain of sexuality, with almost all participants indicating “no need”, which aligns with the findings of previous studies conducted in China [25]. The majority of Chinese patients adhere to conservative cultural norms and are hesitant to disclose information regarding sexual behaviors. Additionally, patients may not prioritize sexual needs as highly as psychological and physical needs.

### Factors associated with the SCNs of cancer patients

Educational background significantly influences health system and information needs, with higher educational levels correlating with dissatisfaction with available information. Individuals with higher levels of education tend to actively seek more information and treatment options. Studies have shown that cancer patients with lower educational levels are less likely to receive clear health information [40]. Despite having lower information needs, there remains a gap in understanding for these patients in terms of health-related information [41]. Effective communication between healthcare providers and patients is essential, especially for individuals with lower educational levels and limited health literacy.

Sex plays a significant role in SCNs, with females often expressing greater psychological and physical needs, which is consistent with previous research findings [38,42]. Females tend to express their psychological and physical needs more openly. Furthermore, the results of this study suggested that age does not significantly impact SCNs. Various studies suggest that younger patients have more needs, such as higher life expectations, increased reliance on family or healthcare providers, elevated psychological needs, and greater patient care and support needs during treatment [10,25].

### Relationship between unmet SCNs and QoL in cancer patients

This study revealed a correlation between unmet SCNs in cancer patients and a decline in QoL. The care needs of cancer patients negatively predict their QoL. The higher the level of demand is, the lower the QoL is, which is consistent with previous research [11]. In this study, the dimensions of physical and daily living needs were most closely associated with QoL, which is consistent with the results of a study in Singapore [43]. The reason is that although the physical symptoms of cancer patients are controlled after admission to the hospital for treatment, unlike healthy individuals, they still suffer from the side effects of treatment. Maslow stated in the hierarchical theory of basic human needs that physiological needs are the most basic, strongest, and most beneficial needs of humankind, and they are the basis for other needs. If a patient’s physiological needs are not met, then his or her QoL will not be guaranteed [31]. In addition, in this study, the dimension of psychological needs was also closely related to QoL. If the patient’s physical needs are met, there will be higher-level needs. Psychological and emotional needs are among these unmet needs. The relationship between SCNs and QoL emphasizes the importance of regularly evaluating cancer patients’ needs and implementing comprehensive interventions [44]. Addressing SCNs is fundamental for successful interventions, benefiting both QoL and psychosocial outcomes [45]. Therefore, healthcare providers should conduct regular and thorough assessments of SCNs and QoL, offering appropriate psychological, physical, and social support to help patients alleviate physical symptoms, reduce psychological distress, enhance cognition, and improve their QoL.

### Implications for nursing practice and research

This study highlights the importance of healthcare providers prioritizing and regularly evaluating care needs and addressing unmet SCNs to improve patient well-being. The findings of this study could help healthcare providers better understand the needs of patients and establish a structure for tailored interventions aimed at managing the various SCNs experienced by cancer patients. Assisting patients in improving their functional levels across various domains can ultimately improve their QoL and treatment outcomes.

### Limitations

This research has various limitations that should be acknowledged. First, this was a single‐center, cross‐sectional study with a small number of participants. Selection bias may occur due to convenience sampling. To increase sample diversity and representativeness, we included patients of various ages, sexes, disease types, and treatment stages. A small number of illiterate patients required assistance in completing the questionnaire, potentially introducing response bias. To mitigate this, we carefully verified their responses to minimize bias and ensure data accuracy. The outcomes predominantly reflect the preferences of participants in China and may not apply to high-income regions or nations. Subsequent research should focus on prospective multicenter studies to explore the extent to which QoL reaches a level comparable to that of the general population and the timing when this occurs.

## Conclusions

This research examined the correlation between unmet SCNs and QoL among cancer patients in China. The findings indicate that a notable proportion of cancer patients experience elevated unmet needs, primarily in the health system and information domain, followed by the psychological, physical and daily living domains. Various factors, such as sex, education level, disease awareness, smoking status, and drinking status, significantly impair the QoL of cancer patients. Healthcare providers should strengthen their skills in observation and communication to evaluate patient needs more effectively and facilitate emotional expression. Additionally, the implementation of more efficient interventions and the provision of high-quality supportive care are essential for addressing these needs and enhancing the QoL of cancer patients.

## Supporting information

S1 Table Comparison of the quality of life of participants with met and unmet needs. (DOCX)

S1 Checklist STROBE Statement—Checklist of items that should be included in reports of cross-sectional studies. (DOC)
