# Supplementary material for: Supportive care needs and quality of life among cancer patients in China: A cross-sectional study
Source: PLoS One. 2025 Aug 28;20(8):e0331149. doi: 10.1371/journal.pone.0331149 (PMC12393739; doi:10.1371/journal.pone.0331149)
Supplement: S1 Table — (DOCX) [file pone.0331149.s001.docx]

**S1 Table. Comparison of the quality of life of participants with met and unmet needs.**

| QoL | Physical function | | Role function | | Cognitive function | | Emotional function | | Social function | | Global QOL | |
| --- | --- | --- | --- | --- | --- | --- | --- | --- | --- | --- | --- | --- |
| SCNs | M (SD) | *p* | M (SD) | *p* | M (SD) | *p* | M (SD) | *p* | M (SD) | *p* | M (SD) | *p* |
| PSY |  |  |  |  |  |  |  |  |  |  |  |  |
| Met | 76.28 (21.98) | **0.007** | 63.62 (21.13) | **0.011** | 90.70 (15.89) | **0.001** | 86.62 (17.28) | **<0.001** | 66.35 (16.74) | **0.001** | 72.28 (16.84) | **0.002** |
| Unmet | 62.83 (32.02) |  | 51.52 (30.15) |  | 78.79 (20.53) |  | 54.04 (28.95) |  | 53.54 (24.57) |  | 60.35 (25.26) |  |
| HSI |  |  |  |  |  |  |  |  |  |  |  |  |
| Met | 76.03 (20.63) | 0.107 | 63.93 (20.57) | 0.068 | 89.66 (16.73) | 0.161 | 83.54 (21.64) | **0.008** | 67.30 (17.59) | **0.005** | 71.41 (16.59) | 0.166 |
| Unmet | 68.97 (30.27) |  | 56.32 (27.72) |  | 85.34 (19.01) |  | 72.27 (27.59) |  | 57.76 (20.99) |  | 66.67 (23.31) |  |
| PDL |  |  |  |  |  |  |  |  |  |  |  |  |
| Met | 77.02 (21.45) | **<0.001** | 63.89 (21.26) | **<0.001** | 91.67 (12.98) | **<0.001** | 82.90 (21.89) | **<0.001** | 66.67 (15.36) | **<0.001** | 73.32 (16.71) | **<0.001** |
| Unmet | 53.33 (33.27) |  | 44.93 (30.75) |  | 68.84 (25.28) |  | 58.33 (28.98) |  | 46.38 (28.41) |  | 50.00 (22.61) |  |
| PCS |  |  |  |  |  |  |  |  |  |  |  |  |
| Met | 73.50 (23.97) | 0.563 | 60.61 (22.57) | 0.895 | 88.70 (17.38) | 0.116 | 81.20 (22.89) | **0.001** | 63.23 (18.99) | 0.952 | 69.90 (19.07) | 0.418 |
| Unmet | 69.58 (34.59) |  | 61.46 (34.28) |  | 81.25 (20.07) |  | 60.42 (31.84) |  | 63.54 (24.51) |  | 65.63 (24.88) |  |
| SEX |  |  |  |  |  |  |  |  |  |  |  |  |
| Met | 73.33 (25.26) | 0.269 | 61.11 (23.92) | 0.105 | 88.15 (17.26) | 0.090 | 79.20 (24.62) | 0.100 | 63.46 (18.72) | 0.337 | 70.06 (18.92) | **0.001** |
| Unmet | 53.33 (28.28) |  | 33.34 (23.57) |  | 66.67 (47.14) |  | 50.00 (35.36) |  | 50.00 (70.71) |  | 25.00 (35.36) |  |

**S1 Table.** (Continued)

| QoL | Dyspnea | | Pain | | Fatigue | | Insomnia | | Appetite loss | | Nausea/Vomiting | |
| --- | --- | --- | --- | --- | --- | --- | --- | --- | --- | --- | --- | --- |
| SCNs | M (SD) | *p* | M (SD) | *p* | M (SD) | *p* | M (SD) | *p* | M (SD) | *p* | M (SD) | *p* |
| PSY |  |  |  |  |  |  |  |  |  |  |  |  |
| Met | 12.82 (23.37) | **<0.001** | 9.46 (18.40) | **0.001** | 23.61 (22.15) | **<0.001** | 17.63 (26.26) | **0.001** | 19.23 (27.75) | **0.005** | 8.49 (14.69) | **0.028** |
| Unmet | 31.31 (31.11) |  | 24.24 (33.10) |  | 41.75 (31.68) |  | 38.38 (37.38) |  | 36.36 (35.71) |  | 17.17 (30.19) |  |
| HSI |  |  |  |  |  |  |  |  |  |  |  |  |
| Met | 13.50 (24.76) | 0.052 | 8.65 (18.84) | **0.011** | 26.44 (23.76) | 0.418 | 17.30 (25.52) | **0.016** | 20.67 (28.40) | 0.233 | 10.34 (17.15) | 0.866 |
| Unmet | 22.41 (28.19) |  | 18.97 (27.83) |  | 30.08 (28.54) |  | 29.88 (35.15) |  | 27.01 (33.31) |  | 10.92 (23.06) |  |
| PDL |  |  |  |  |  |  |  |  |  |  |  |  |
| Met | 12.57 (23.21) | **<0.001** | 8.77 (17.77) | **<0.001** | 22.12 (20.40) | **<0.001** | 16.96 (25.18) | **<0.001** | 17.84 (25.54) | **<0.001** | 8.19 (15.24) | **0.001** |
| Unmet | 40.58 (30.08) |  | 34.06 (35.35) |  | 57.01 (30.58) |  | 50.72 (38.76) |  | 50.72 (38.76) |  | 22.46 (32.42) |  |
| PCS |  |  |  |  |  |  |  |  |  |  |  |  |
| Met | 17.08 (25.86) | 0.814 | 12.95 (23.81) | 0.925 | 27.64 (24.81) | 0.673 | 21.21 (29.19) | 0.136 | 22.86 (30.44) | 0.606 | 10.19 (18.80) | 0.527 |
| Unmet | 18.75 (32.13) |  | 13.54 (22.13) |  | 30.56 (33.58) |  | 33.33 (38.49) |  | 27.08 (32.70) |  | 13.54 (26.68) |  |
| SEX |  |  |  |  |  |  |  |  |  |  |  |  |
| Met | 16.54 (26.04) | **0.008** | 11.98 (21.99) | **<0.001** | 27.32 (25.46) | **0.014** | 21.97 (29.97) | **0.039** | 22.72 (30.11) | **0.043** | 10.12 (19.33) | **0.025** |
| Unmet | 66.67 (0.00) |  | 83.34 (23.57) |  | 72.23 (7.86) |  | 66.67 (47.14) |  | 66.67 (47.14) |  | 41.67 (35.36) |  |

**S1 Table.** (Continued)

| QoL | Constipation | | Diarrhea | | Financial difficulties | |
| --- | --- | --- | --- | --- | --- | --- |
| SCNs | M (SD) | *p* | M (SD) | *p* | M (SD) | *p* |
| PSY |  |  |  |  |  |  |
| Met | 8.65 (19.15) | 0.126 | 2.88 (9.42) | **0.025** | 39.42 (24.03) | **0.028** |
| Unmet | 15.15 (26.47) |  | 9.09 (22.47) |  | 50.50 (27.79) |  |
| HSI |  |  |  |  |  |  |
| Met | 8.02 (17.87) | 0.157 | 3.80 (11.92) | 0.569 | 38.40 (24.51) | **0.046** |
| Unmet | 13.22 (24.93) |  | 5.17 (16.28) |  | 47.12 (25.77) |  |
| PDL |  |  |  |  |  |  |
| Met | 7.89 (18.97) | **0.004** | 3.51 (11.19) | 0.103 | 38.89 (23.43) | **0.001** |
| Unmet | 21.74 (27.72) |  | 8.70 (22.96) |  | 57.97 (28.81) |  |
| PCS |  |  |  |  |  |  |
| Met | 10.47 (21.53) | 0.707 | 4.41 (14.23) | 0.948 | 41.60 (24.07) | 0.532 |
| Unmet | 8.33 (19.25) |  | 4.17 (11.38) |  | 45.83 (34.16) |  |
| SEX |  |  |  |  |  |  |
| Met | 9.88 (20.79) | 0.121 | 4.44 (13.99) | 0.655 | 41.73 (25.01) | 0.168 |
| Unmet | 33.34 (47.14) |  | 0.00 (0.00) |  | 66.67 (47.14) |  |

M = Mean

SD = standard deviation

p = p-value; a value below 0.05 is considered to be statistically significant

Abbreviations: QoL: Quality of life; SCNs: Supportive care needs; PSY: Psychological; HIS: Health systems and information; PDL: Physical and daily living; PCS: Patient care and support; SEX: Sexuality
