# Supplementary material for: Supportive care needs and quality of life among cancer patients in China: A cross-sectional study
Source: PLoS One. 2025 Aug 28;20(8):e0331149. doi: 10.1371/journal.pone.0331149 (PMC12393739; doi:10.1371/journal.pone.0331149)
Supplement: S1 Checklist — (DOC) [file pone.0331149.s002.doc]

STROBE Statement—Checklist of items that should be included in reports of ***cross-sectional studies***

|  | Item No | Recommendation |
| --- | --- | --- |
| **Title and abstract** | 1 | (1) Title: Supportive Care Needs and Quality of Life among Cancer Patients in China: A Cross-sectional Study |
|  |  | (2) Abstract: Among 137 Chinese cancer patients, unmet needs (highest in health-system/info) were tied to sex, education, awareness, smoking, drinking, and lower QoL (p<0.05). |
| Introduction | | |
| Background/rationale | 2 | Cancer causes 30% of deaths in China; unmet supportive-care needs (SCNs) lower QoL and worsen outcomes. Nurses are key to identifying and addressing these needs. This study assessed SCNs, their determinants, and impact on QoL in 137 Chinese cancer patients to guide tailored interventions |
| Objectives | 3 | Specify the precise aims of the study, including any hypotheses that were predetermined |
| Methods | | |
| Study design | 4 | Cross-sectional study of 137 cancer inpatients using SCNS-SF34-C and EORTC QLQ-C30 |
| Setting | 5 | July–August 2023, 150 cancer inpatients at a Chinese tertiary hospital were recruited; informed consent, assistance for low-literacy participants, targeting 125–210 based on 21 variables |
| Participants | 6 | 1. Inclusion: ≥18 y, confirmed cancer, consent, communicable  2. Exclusion: major recent stress or mental disorder |
| Variables | 7 | 1. Outcomes: Supportive Care Needs, Quality of life.  2. Predictors: age, gender, occupation, marital status, educational background, disease awareness, monthly income, drinking, and smoking habits.  3. Potential confounders: clinical factors such as disease stage, symptoms, and disease progression. |
| Data sources/ measurement | 8* | 1. Demographic and lifestyle data via questionnaires; cancer type/treatment from EMRs, cross-checked by staff  2. SCNS-SF34-C: 34-item, 5-domain Chinese cancer-needs scale (1–5); ≥3 = unmet. α > 0.7, validated  3. EORTC QLQ-C30: 30-item cancer QoL scale—5 function, 3 symptom, global, 6 single items; higher function/global = better, symptoms = worse; validated. |
| Bias | 9 | Investigators assisted low-literacy/older participants and clinicians verified all data for completeness and consistency |
| Study size | 10 | Among the 150 participants, 137 completed the questionnaires (response rate: 91.3%) |
| Quantitative variables | 11 | t-tests/ANOVA compared SCN groups; Spearman linked SCNs to QoL domains. Unmet = mean ≥ 3. p < 0.05 |
| Statistical methods | 12 | Data were analyzed with SPSS 23.0. Demographics were summarized using counts/% for categorical variables and mean ± SD for continuous variables. Differences in SCNs across demographic and clinical groups were tested with t-tests (two groups) or one-way ANOVA (> two groups). Spearman correlations assessed associations between SCNs and QoL domains. Unmet needs were defined as item means ≥ 3, and QoL was compared between met/unmet groups. Significance was set at p < 0.05 |
| Results | | |
| Participants | 13* | Out of 150 participants, 137 completed the questionnaires (response rate: 91.3%). Nine individuals refused to participate in the survey, while four participants had recently been exposed to other major stressful events. (Fig. 1) |
|  |  | **Fig. 1** Patient flow.  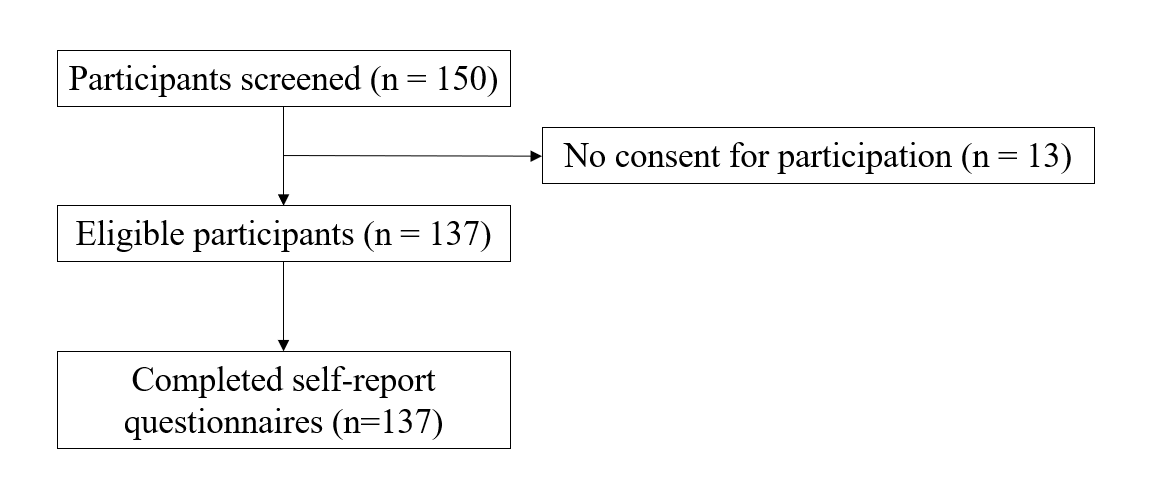 |
| Descriptive data | 14* | Among the 150 participants, 137 completed the questionnaires (response rate: 91.3%). The average age of the participants was 57.48 years (SD = 10.80). In total, 98.54% of the participants were married, and 57.96% had a middle school education. The majority of the participants were farmers (n = 68, 49.64%) and earned an income of less than 1000 Chinese yuan (CNY) per month (n = 58, 42.32%). The most common primary cancer types were digestive system cancer (n = 50, 36.50%) and lung cancer (n = 44, 32.12%) |
| Outcome data | 15* | Among 137 Chinese cancer patients, the overall SCNS-SF34-C mean score was 2.34 (SD 0.55). The health-system/info domain scored highest (M = 2.74), followed by psychological (2.36) and sexuality lowest (1.10). The three top unmet items were “one staff contact” (3.43), “treated as a person” (3.28) and “access to counseling” (3.26) |
| Main results | 16 | Following the method of the previous study, unmet needs were defined as item mean scores of 3 or higher, and the study compared QoL between groups with met and unmet needs. Individuals with unmet psychological as well as physical and daily living needs show lower scores in all functions (p < 0.01) and global QoL (p < 0.01), along with higher scores in nearly all symptom domains (all p < 0.05), except for constipation and nausea/vomiting. Specific items such as pain (p = 0.011), insomnia (p = 0.016), and financial difficulties (p = 0.046) were observed in individuals with unmet needs in the health system and information domain, who also reported poorer emotional (p = 0.008) and social (p = 0.005) function. Unmet patient care and support needs were associated with impaired emotional function (p = 0.001), while unmet sexual needs were associated with increased dyspnea (p = 0.008), pain (p < 0.001), fatigue (p = 0.014), insomnia (p = 0.039), appetite loss (p = 0.043), and nausea/vomiting (p = 0.025) |
| Other analyses | 17 | Factors such as gender (p = 0.047), educational background (p = 0.042), disease awareness (p = 0.016), smoking (p = 0.027), and alcohol (p = 0.033) consumption were found to be associated with unmet SCNs across various domains including physical and daily living, health system and information, psychological, and sexuality |
| Discussion | | |
| Key results | 18 | Chinese cancer patients face notable unmet needs in the health system and information domain. Future studies should focus on designing individualized interventions to improve supportive care among cancer patients and to enhance their QoL |
| Limitations | 19 | Limitations: single-center, cross-sectional design with 137 patients selected by convenience, risking selection and response bias (illiterate participants needed assistance). Findings may not generalize beyond China. Larger, prospective multicenter studies are warranted to track QoL recovery and generalizability |
| Interpretation | 20 | This research examines the correlation between unmet SCNs and the QoL among cancer patients in China. Findings indicate a notable proportion of cancer patients experience elevated unmet needs primarily in the health system and information domain, followed by the psychological, as well as the physical and daily living domain. Various factors such as gender, education level, disease awareness, smoking, and drinking significantly impair the QoL of cancer patients |
| Generalisability | 21 | This research underscores the importance of healthcare providers prioritizing and continuously assessing the care needs of cancer patients, particularly addressing any unmet SCNs to enhance patient well-being. The study's insights can guide healthcare professionals in understanding patient needs and developing personalized interventions to manage the diverse SCNs encountered by individuals with cancer. By aiding patients in enhancing their functional capacities across different domains, overall QoL and treatment outcomes can be significantly improved |
| Other information | | |
| Funding | 22 | This work was supported by the Health and Family Planning Commission of Hebei  Province, China, (Grant No. 20211424) |

*Give information separately for exposed and unexposed groups.

**Note:** An Explanation and Elaboration article discusses each checklist item and gives methodological background and published examples of transparent reporting. The STROBE checklist is best used in conjunction with this article (freely available on the Web sites of PLoS Medicine at http://www.plosmedicine.org/, Annals of Internal Medicine at http://www.annals.org/, and Epidemiology at http://www.epidem.com/). Information on the STROBE Initiative is available at www.strobe-statement.org.
